# Topoisomerase I as a Biomarker: Detection of Activity at the Single Molecule Level

**DOI:** 10.3390/s140101195

**Published:** 2014-01-10

**Authors:** Joanna Proszek, Amit Roy, Ann-Katrine Jakobsen, Rikke Frøhlich, Birgitta R. Knudsen, Magnus Stougaard

**Affiliations:** 1 Department of Pathology, Aarhus University Hospital, Aarhus C 8000, Denmark; E-Mails: joanna.proszek@gmail.com (J.P.); annkatrinejakobsen@gmail.com (A.-K.J.); 2 Department of Molecular Biology and Genetics, Aarhus University, Aarhus C 8000, Denmark; E-Mails: royamit79@yahoo.com (A.R.); rff@mb.au.dk (R.F.); brk@mb.au.dk (B.R.K.); 3 Department of Biotechnology, National Institute of Pharmaceutical Education & Research (NIPER), Hajipur-844 101, Bihar, India

**Keywords:** Topoisomerase-I, camptothecin, enzyme activity, biosensor, tyrosyl-DNA phosphodiesterase 1, single molecule, drug response, cancer

## Abstract

Human topoisomerase I (hTopI) is an essential cellular enzyme. The enzyme is often upregulated in cancer cells, and it is a target for chemotherapeutic drugs of the camptothecin (CPT) family. Response to CPT-based treatment is dependent on hTopI activity, and reduction in activity, and mutations in hTopI have been reported to result in CPT resistance. Therefore, *hTOPI* gene copy number, mRNA level, protein amount, and enzyme activity have been studied to explain differences in cellular response to CPT. We show that Rolling Circle Enhanced Enzyme Activity Detection (REEAD), allowing measurement of hTopI cleavage-religation activity at the single molecule level, may be used to detect posttranslational enzymatic differences influencing CPT response. These differences cannot be detected by analysis of hTopI gene copy number, mRNA amount, or protein amount, and only become apparent upon measuring the activity of hTopI in the presence of CPT. Furthermore, we detected differences in the activity of the repair enzyme tyrosyl-DNA phosphodiesterase 1, which is involved in repair of hTopI-induced DNA damage. Since increased TDP1 activity can reduce cellular CPT sensitivity we suggest that a combined measurement of TDP1 activity and hTopI activity in presence of CPT will be the best determinant for CPT response.

## 1. Introduction

DNA-modifying enzymes have been used for many years as drug targets in chemotherapeutic anticancer therapy, which exploits the high transcription and replication rates of cancer cells. As a consequence there has been a growing interest in using genetic and bio-enzymatic information related to these enzymes to predict drug response. An example of DNA-modifying enzymes that are targeted by chemoterapeutic agents is the human enzyme, topoisomerase I (hTopI). hTopI is an essential nuclear enzyme which releases the topological stress resulting during processes such as transcription and replication, where the two DNA strands in the DNA double helix are locally unwound. Enzymatically, hTopI acts through the transient cleavage and subsequent religation of one strand of the DNA helix [1]. The enzyme is overexpressed in a wide range of cancers [2] and it is the sole cellular target of anticancer drugs from the camptothecin (CPT) family mainly used in systemic treatment of colon-, ovarian- and small cell lung cancer [3–5]. Recently drugs of the CPT family have also been used for treatment of upper gastrointestinal-, cervical-, and pancreatic cancer [6–9]. CPT exhibits its toxicity by intercalating between the bases of the DNA in the hTopI-induced nicks and is stabilized through interactions to both the DNA and hTopI [10]. CPT poisons the cells mainly through the generation of double-stranded DNA breaks caused by S-phase specific collision of replication forks with the hTopI-DNA complexes [11]. However, CPT also damages non-dividing cells through collision of the complexes with DNA repair processes and transcription forks [12]. Stabilization of hTopI-DNA complexes may also occur as the result of cleavage near endogenous DNA lesions (such as nicks, abasic sites, or mismatches) [13,14] but are normally promptly repaired, e.g., through the tyrosyl-DNA phosphodiesterase 1 (TDP1) and Poly(ADP-ribose) polymerase (PARP) dependent pathway [15]. Thus, enzymatic factors other than hTopI influence the patient response rate for CPT-based treatment, which for CPT monotherapy is around 20%–30%, but may be increased to a response rate of around 50% in combination with other agents [16–18].

hTopI has been widely evaluated as a predictive biomarker for CPT-based therapy both at gene-, mRNA-, protein-, and activity level with somewhat diverging results. In some studies the gene-copy number of *hTOPI* has been found to correlate with protein expression and with CPT sensitivity [19,20]. In contrast, others have found that neither the mRNA expression nor protein amount of hTopI was predictive for CPT sensitivity whereas hTopI activity correlated with the CPT sensitivity [12,21]. Furthermore, certain mutations in hTopI have been demonstrated to cause CPT resistance [22,23]. We show here that direct determination of the drug response of hTopI is a better predictive marker for cellular CPT sensitivity than looking solely at gene copy number, mRNA amount, protein amount, or hTopI activity without drug. Furthermore, since other factors than hTopI have been shown to influence CPT response we suggest that additional assays, e.g., measurement of TDP1 activity may be included.

## 2. Experimental Section

### 2.1. Reagents and Enzymes

T4 polynucleotide kinase, Phi29 DNA polymerase, T4 DNA ligase, exonuclease I (ExoI), and exonuclease III (ExoIII) were obtained from Fisher Scientific (Slangerup, Danmark). All oligonucleotides were obtained from DNA Technology A/S (Aarhus, Denmark). CodeLink Activated Slides came from SurModics (Eden Prairie, MN, USA), and Vectashield was from Vector Laboratories (Peterborough, UK). Pap Pen was purchased from Dako (Glostrup, Denmark), CPT was from Sigma-Aldrich (Broenby, Denmark). Cell culture media (Minimum Essential Medium and McCoy 5A medium), Fetal Bovine Serum (FBS), 0.25% Trypsin-EDTA (25200-056), Non-Essential Amino Acid (11140-050) and PenStrep (15140-122) stock were obtained from Invitrogen (Naerum, Denmark).

### 2.2. Substrates, Primers and Probes

The substrate for hTopI, S(hTopI)Id16, had the sequence 5′-AGA AAA ATT TTT AAA AAA ACT GTG AAG ATC GCT TAT TTT TTT AAA AAT TTT TCT AAG TCT TTT AGA TCC CTC AAT GCT GCT GCT GTA CTA CGA TCT AAA AGA CTT AGA-3′, the positive control substrate, S(PosC)Id33, had the sequence 5′-p-AGA AAA ATT TTT AAA AAA ACT GTG AAG ATC GCT TAT TTT TTT AAA AAT TTT TCT AAG TCT TTT AGA TCC CTC AAT GCA CAT GTT TGG CTC CGA TCT AAA AGA CTT-3′, the fluorescently labeled detection oligonucleotides, ID16-TAMRA and ID33-6FAM, had the sequences 5′-TAMRA-CCT CAA TGC TGC TGC TGT ACT AC-3′ and 5′-6FAM-CCT CAA TGC ACA TGT TTG GCT CC-3′ respectively. The Rolling Circle Amplification (RCA) primer used for Rolling Circle Enhanced Enzyme Activity Detection (REEAD) had the sequence 5′-C6amine-CCA ACC AAC CAA CCA AAT AAG CGA TCT TCA CAG T-3′. The TDP1-biosensor had the sequence 5′-ATTO488-AAA GCA GGC TTC AAC GCA ACT GTG AAG ATC GCT TGG GTG CGT TGA AGC CTG CTT T-BHQ1-3′.

### 2.3. Cell Culture and Extract Preparation

Caco2 cells were grown in minimum essential medium (MEM) supplemented with 20% FBS, 1% non-essential amino acids, 1% PenStrep. HT29 cells were grown in McCoy 5A medium supplemented with 10% FBS, 1% PenStrep. The cell cultures were maintained in a humidified incubator (5% CO_2_/95% air atmosphere at 37 °C). Cells were harvested by trypsin treatment (0.25% Trypsin-EDTA solution). The trypsin was inactivated with FBS-containing media, followed by two consecutive washes with 1× PBS. Cells were counted, aliquoted into tubes each containing 5 × 10^5^ cells, and stored at −80 °C until further analysis. Preparation of whole cell extracts was done by mixing 5 × 10^5^ cells with 500 μL, 250 μL, or 100 μL lysis buffer (10 mM Tris-HCl, pH 7.5, 5 mM EDTA, 1 mM phenylmethylsulfonyl fluoride, and 1 mM DTT) for hTopI activity measurement, hTopI activity measurement in the presence of CPT, and TDP1 activity measurement, respectively. Tubes with cells and lysis buffer were incubated on ice for 10 min before the extract was used.

### 2.4. Western Blot Analysis of hTopI in Cell Extracts

Cell extracts from Caco2 or HT29 cells were analyzed by 10% SDS-polyacrylamide gel-electrophoresis followed by western blotting onto a Nitrocellulose Protran BA85 (GE Healthcare Life Sciences, Broenby, Denmark) membrane and antibody incubation using a polyclonal hTopI antibody (Topogen, Port Orange, FL, USA) and a Anti-TATA binding protein TBP [1TBP18] antibody (Abcam, Cambridge, UK) as loading control.

### 2.5. Detection of hTopI Activity by REEAD Assay

The hTopI reaction was carried out in a 20 μL reaction volume containing a divalent cation depletion buffer (10 mM Tris-HCl, pH 7.5, 5 mM EDTA, 50 mM NaCl, 0.5 mM phenylmethylsulfonyl fluoride, and 0.5 mM DTT) supplemented with S(hTopI)Id16 DNA substrate to a final concentration of 0.5 μM. Reactions were initiated by the addition of 10 μL cell extract in the presence or absence of CPT as indicated in the figure legends. DMSO was added as solvent control of CPT. Incubation was continued at 37 °C for 60 min for the dilution experiments and 15 min for CPT sensitivity experiments, before the heat inactivation of the enzymes by incubation for 5 min at 95 °C. To allow quantification, 5 nM of ligated S(PosC)Id33 was added to the heat inactivated reaction mixture and to increase the efficiency of hybridization NaCl was added to a final concentration of 250 mM. Preparation of the ligated S(PosC)Id33 was done using the T4 DNA ligase, and the resulting ligated circles were exonuclease digested with ExoI and ExoIII to remove non-ligated S(PosC)Id33 before gel-purification using 8% polyacrylamide gel. The concentration of the obtained circles was determined by spectrophotometric measurement. The 5′-amine-coupled RCA primer was linked to CodeLink Activated Slides according to the manufacturer's protocol. Subsequently, reaction mixtures were hybridized to the surface coupled primer for 60 min at 37 °C. Slides were washed for 1 min at room temperature in wash buffer 1 (100 mM Tris-HCl, pH 7.5, 150 mM NaCl, and 0.3% SDS) and subsequently for 1 min at room temperature in wash buffer 2 (100 mM Tris-HCl, pH 7.5, 150 mM NaCl, and 0.05% Tween-20). Finally, the slides were dehydrated in 99.9% ethanol for 1 min and air-dried. Rolling circle DNA synthesis was performed for 60 min at 37 °C in 1× Phi29 buffer supplemented with 0.2 μg/μL BSA, 250 μM dNTP, and 1 unit/μL Phi29 DNA polymerase. The reaction was stopped by washing in wash buffers 1 and 2 for 1 min each followed by dehydration in 99.9% ethanol for 1min. The RCA products were detected by hybridization of 0.2 μM of each of the detection probes ID16-TAMRA (red fluorescent labeled) and ID33-6FAM (green fluorescent labeled) in a buffer containing 20% formamide, 2× SSC, and 5% glycerol overnight at 37 °C. The slides were washed in wash buffer 1 for 10 min, in wash buffer 2 for 5 min, dehydrated in ethanol for 1 min, mounted with Vectashield, and visualized under a fluorescence microscope as described previously [24]. For quantitative depiction of the results, the number of signals originating from hTopI activity (red signals) or spike in control circles (green signals), respectively, were counted on 10 pictures and the results calculated as the ratio between the number of red (R) and green (G) signals (R/G) as described [24]. All experiments were done in triplicate

### 2.6. Survival Assay

The cells were seeded at 6 × 10^4^ cells/cm^2^ and allowed to adhere before CPT was added to the media at final concentrations of 0.1, 0.2, 0.5, and 1 μM. CPT-treatment was continued for 24 h at 5% CO_2_/95% air atmosphere at 37 °C. After harvesting as described above, cells were resuspended in 1× PBS, and the proportions of dead and live cells were determined by trypan blue staining (0.4%) followed by cell counting in a hemocytometer. The viability was calculated using the formula (live cell count/total cell count) × 100.

### 2.7. TDP1 Activity Assay

A dilution series of 5 × 10^5^, 2.5 × 10^5^, and 1.25 × 10^5^ cells/100 μL was made using lysis buffer as diluent. The TDP1 activity assay was carried out in a black Corning 384 well microplate with a final volume of 25 μL containing 20 μL cell extract, 1× TDP-buffer (20 mM Tris-HCl pH 8, 100 mM KCl, 10 mM DTT, 10 mM EDTA and 0.05% Triton X-100), and 0.5 μM TDP1-biosensor. For the negative control lysis buffer was used instead of cell extract. Real-time fluorescence measurements were performed every 10 s at 37 °C using a FlexStation 3 set to an excitation wavelength at 494 nm, an emission wavelength at 518 nm and a cutoff at 515 nm. The data were transferred to Microsoft Excel, where the linear slope from the data measured from 15 to 20 min was calculated for all samples and thereafter visualized as an XY-plot using GraphPad Prism. The experiments were done in triplicate.

## 3. Results and Discussion

### 3.1. Correlation of hTOPI Gene Copy Number and Protein Amount with Enzyme Activity

Although hTopI has been examined previously for use as a predictive biomarker of CPT sensitivity these studies have focused solely on the level of gene copy number, mRNA expression, protein amount, or activity rather than testing the CPT response of the endogenous expressed hTopI. The *hTOPI* gene copy number is frequently increased in colorectal cancer [25,26] and the two colon cancer cell lines used here, Caco2 and HT29 are known to have an approximate twofold difference in *hTOPI* gene copy number. Caco2 has eight copies of the *hTOPI* gene, whereas HT29 has five [27]. These cell lines were examined with regards to hTopI protein amount, hTopI activity, cellular CPT sensitivity, TDP1 activity, and CPT response of hTopI in whole cell extracts.

**Figure 1.**
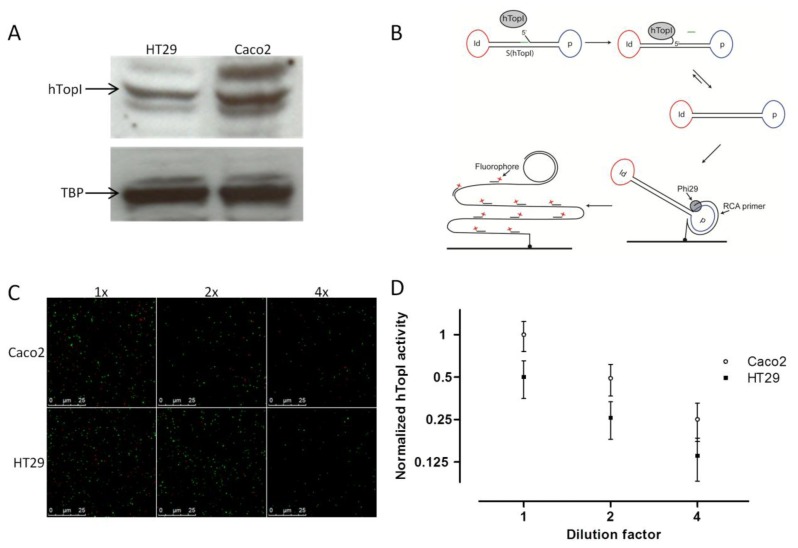
(**A**) Western blot comparing the amount of hTopI protein in HT29 and Caco2. TATA binding protein (TBP) was used as loading control. (**B**) Graphic depiction of the REEAD assay. S(hTopI) is designed to adopt a dumbbell structure consisting of a stretch of double-stranded DNA connected by two loops holding a sequence identifying the substrate (marked Id and depicted in red) and a primer binding sequence (marked p and depicted in blue). The double-stranded DNA has a preferred recognition sequence for hTopI that enables hTopI to cleave S(hTopI) releasing three bases of DNA (shown in green) from the 3′end of S(hTopI). This enables the 5′end of S(hTopI) to hybridize, and hTopI can subsequently ligate the substrate into a closed circle. The generated circle is hybridized to a surface-attached primer matching the primer binding sequence (indicated by p). Subsequently, Phi29 DNA polymerase mix is added to support RCA and the resulting RCA products are visualized by hybridization of fluorescently labeled probes (matching the Id sequence) and the products analyzed using a fluorescence microscope. (**C**) REEAD based detection of hTopI activity in 1x, 2x, and 4x dilution of whole cell extract of Caco2 and HT29. One example (Raw data) randomly picked out of 30 individual microscopic images of each triplicate reaction sample is shown. Red signals represent RCA products generated from circularized S(hTopI) (*i.e.*, generated by hTopI activity) and green signals represent RCA products generated from control circles. (**D**) REEAD based quantification of hTopI activity in 1x, 2x and 4x dilution of whole cell extract of Caco2 and HT29, depicted as mean ±SD in a log2 XY-plot. The data were collected from triplicate reactions and normalized to the mean hTopI activity in undiluted whole cell extract from the Caco2 cell culture.

Similar to McLeod *et al.* [19], we found a correlation between the known *hTOPI* gene copy number and the hTopI protein amount based on western blot analysis of extract from the two cell lines. Thus, the Caco2 cell line, which has eight copies of the *hTOPI* gene, had around a twofold higher amount of hTopI protein compared to the HT29 cell line, which has five copies of the *hTOPI* gene (Figure 1A). This could indicate that there is a similar expression from the *hTOPI* genes in the two cell lines. However, *hTOPI* gene copy number, RNA level, and protein amount is not capable of predicting changes in activity due to posttranslational factors. This may be problematic since the activity of TopI is known to be influenced by posttranslational modifications [28,29] as well as protein-protein interactions [30].

We have previously published a method for highly sensitive detection of hTopI using Rolling Circle Enhanced Enzyme Activity Detection (REEAD), capable of measuring hTopI cleavage-religation activity at the single molecule level (Figure 1B) [24]. This method, which is based on a DNA-sensor with a preferred DNA binding site of hTopI, can detect any changes in activity whether it is DNA-binding, -cleavage, or -ligation. Using this method we found an approximate twofold higher hTopI activity in the extract from the Caco2 cell line compared to the extract from the HT29 cell line (Figures 1C,D). In contrast to previous findings where hTopI protein amount and enzyme activity did not correlate [12,21], we found that the twofold higher hTopI activity in Caco2 correlated well with the *hTOPI* gene copy number and protein amount. This discrepancy between the two studies may likely be due to the use of different cell lines, which regulate hTopI activity differently at the posttranslational level, thus without affecting the hTopI protein amount.

### 3.2. Presence of Gene Mutations in hTOPI or Different TDP1 Activity in Caco2 and HT29

**Figure 2.**
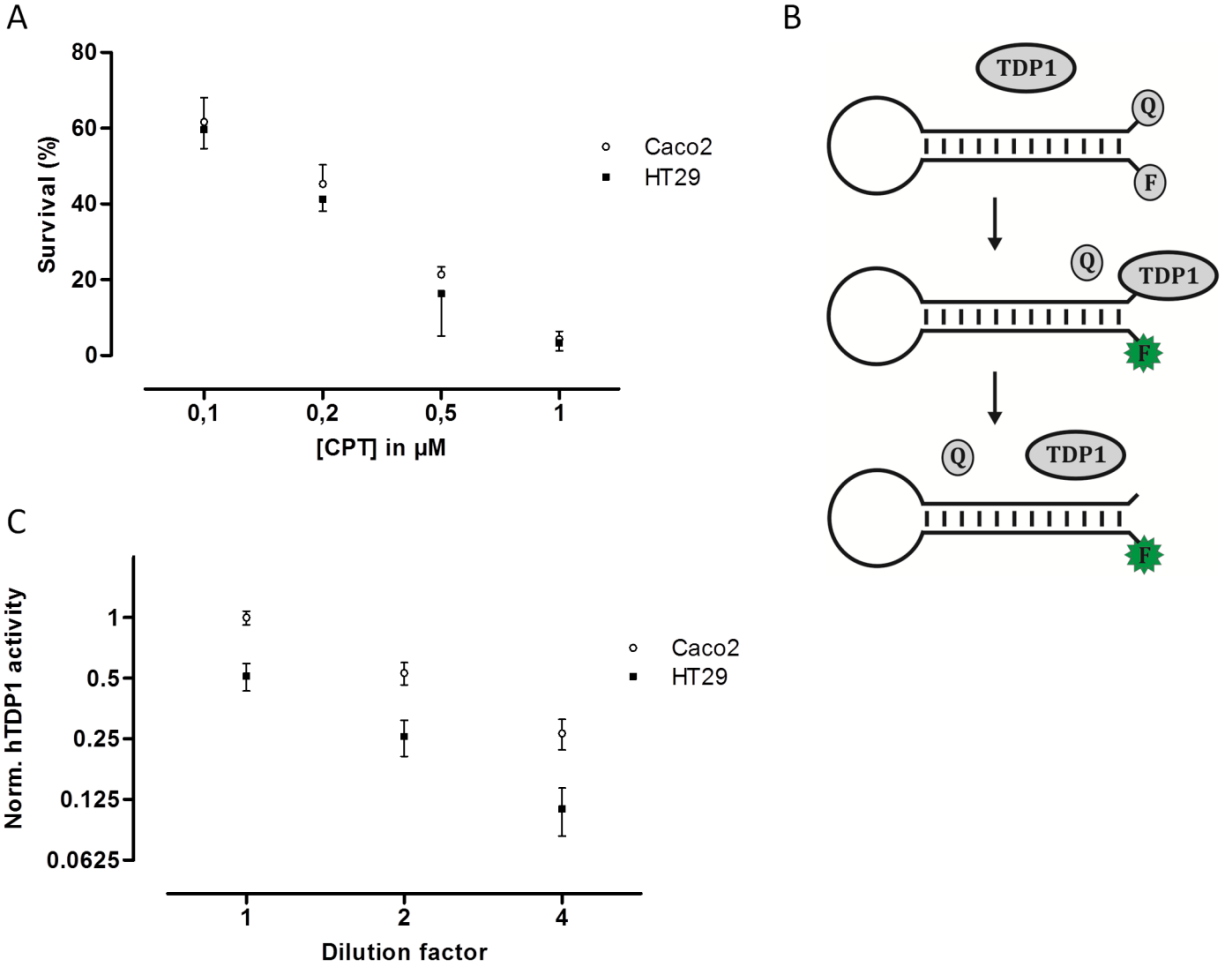
(**A**) Survival assay of Caco2 and HT29 cells in the presence of CPT. Caco2 and HT29 cells were incubated with 0.1, 0.2, 0.5, 1 μM of CPT for 24 hours before the percentage of viable cells was estimated using the trypan blue exclusion method. All data are presented as the percentage of live cells with mean ±SD from triplicate experiments. (**B**) Graphic depiction of the TDP1 assay. The TDP1-biosensor is designed to adopt a beacon structure, bringing the quencher (Q) and fluorophore (F) in proximity thus quenching the emission from the fluorophore. In presence of active TDP1, the quencher is removed from the TDP1-biosensor, and the emission of the fluorophore becomes quantifiable. (**C**) TDP1 activity measurement in 1x, 2x, and 4x dilution of whole cell extract of Caco2 and HT29, depicted as mean ±SD in a log2 XY-plot. The data were collected from triplicate reactions and normalized to the mean TDP1 activity in undiluted whole cell extract from the Caco2 cell culture.

To examine if the *hTOPI* gene copy number, protein amount, or enzyme activity could be correlated to cellular CPT sensitivity, the Caco2 and HT29 cultures were exposed to increasing concentration of CPT and the surviving cells were counted and scored as the percentage of the total number of cells. Despite the fact that the Caco2 cells have eight copies of the *hTOPI* gene, and the HT29 have five, twofold more hTopI protein, and twofold higher activity of hTopI, the Caco2 and HT29 cells had similar sensitivity to CPT (Figure 2A). This was unexpected since hTopI activity had been found to be the best determinant for CPT sensitivity, where high hTopI activity resulted in high cellular CPT sensitivity [21].

An explanation to this could be mutations in the *hTOPI* gene, which have been shown to be able to modify the cellular CPT sensitivity [22,31,32]. However, sequencing of the *hTOPI* genes from either culture did not reveal any mutations in the genes (data not shown). Decreased cellular CPT-sensitivity can be obtained also through upregulation of enzyme activities involved in repair of TopI-DNA complexes such as TDP1 [33,34]. We have previously published an optical DNA-based sensor (Figure 2B) capable of detecting the activity of TDP1 specifically even in crude cellular extract [35]. The TDP1-sensor was shown to be capable of precisely detecting changes in TDP1 activity, and since it has been shown that increased TDP1 activity can influence cellular toxicity of CPT, we measured the activity of TDP1 in extract from both Caco2 and HT29. In Figure 2C it can be seen that Caco2 cells did indeed have an approximate twofold higher TDP1 activity compared to HT29. The higher activity of TDP1 in Caco2 might contribute to the lower CPT sensitivity. Although Barthelmes *et al.* did not measure the TDP1 activity when they showed that overexpression of TDP1 could reduce CPT sensitivity, they reported a more than 100-fold increase in TDP1 protein [33]. Despite that it has been shown that the amount of TDP1 protein does not always correspond to TDP1 activity [36], a 100-fold increase in TDP1 protein is likely to cause much more than a twofold increase in activity. Thus, the twofold increased TDP1 activity in Caco2 compared to HT29 is likely to contribute to the overall CPT sensitivity of the cell line but might not be the only factor.

### 3.3. Posttranslational Modification Changing CPT Sensitivity

**Figure 3.**
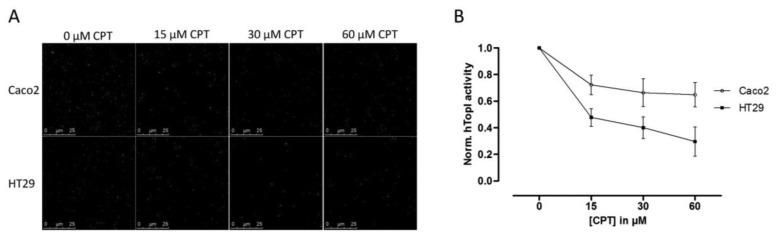
(**A**) REEAD based detection of hTopI activity in whole cell extract of Caco2 and HT29 in the presence of CPT. One example (Raw data) randomly picked out of 30 individual microscopic images of each triplicate reaction sample is shown, red signals represent RCA products generated from circularized S(hTopI) (*i.e.*, generated by hTopI activity) and green signals represent RCA products generated from control circles. (**B**) REEAD based quantification of CPT induced inhibition of hTopI activity in whole cell extract of Caco2 and HT29, depicted as mean ± SD in a log2 XY-plot. The data were collected from triplicate reactions, and each triplicate was normalized to the sample without CPT (thus DMSO only) so that the hTopI activity in the sample without CPT was set to one.

Since hTopI activity has been reported to be regulated both by posttranslational changes [37,38] and protein-protein interactions [30], it is possible that these factors may affect the drug response of hTopI directly. Such factors will not be revealed by measuring hTopI activity, -expression, -gene copy number, or by analyzing the *hTOPI* gene for mutations. Therefore, we speculated that since the activity of the endogenously expressed hTopI did not correlate with the CPT sensitivity of the cell lines, different posttranslational modifications or protein-protein interactions could affect hTopI differently in the two cell lines resulting in similar cellular sensitivity to CPT. We have previously stressed the importance of using sensor systems resembling the natural substrates for the targeted enzymes as much as possible, *i.e.*, DNA-based sensor systems for DNA-modifying enzymes, since this will enable detection of differences in the DNA interaction of both enzymes and drugs [39]. In the REEAD assay such a DNA-based sensor system is exploited and is capable of detecting the cleavage-religation activity of hTopI at the single molecule level. Thus, CPT will prevent religation of the DNA-sensor leading to the generation of fewer closed DNA circles and thereby also fewer signals. Performing REEAD in the presence of CPT should therefore allow detection of any changes in the sensitivity of hTopI towards CPT. As seen in Figure 3 hTopI from the Caco2 cell line was less sensitive towards CPT than was hTopI from HT29 cells. The specificity of REEAD and the use of a divalent cation depleted buffer, leave most enzymes inactive due to lack of co-factors. Thus, it seems plausible that the different drug response observed in the two cell cultures could be a combination of increased TDP1 activity and different drug sensibility of the endogenously expressed hTopI.

## 4. Conclusions and Outlook

Accurate prediction of a response to chemotherapeutic drugs such as CPT, which exert their cytotoxic effect via inhibition of specific enzymatic activities such as hTopI is complicated. There are several factors influencing the cellular response to CPT treatment, including the level of hTopI activity, genetic mutations in the *hTOPI* gene, repair mechanisms such as TDP1, and posttranslational factors which may affect the activity or drug sensibility of hTopI. When comparing Caco2 and HT29, we observed that the Caco2 cell line containing close to twofold the amount of *hTOPI* genes compared to HT29 also had an approximate twofold increase in protein amount and hTopI activity. Since the level of hTopI activity previously has been found to be the best predictor of CPT sensitivity, the Caco2 cells were expected to be more sensitive to CPT than HT29. However, the two cell lines showed identical sensitivity to CPT, something that could not be explained from mutations in the *hTOPI* gene.

Since an increased level of TDP1 has been shown to result in decreased CPT sensitivity, we measured the activity of TDP1 in both the Caco2 and the HT29 cell lines and found an approximate twofold higher TDP1 activity in extract from the Caco2 cells compared to HT29 cells. Although other enzymes are required for repair of hTopI-induced damages in the TDP1-PARP dependent pathway, this difference may contribute to the higher CPT resistance in the Caco2 cell line. However, the twofold increase in TDP1 activity in Caco2 cells compared to HT29 cells is unlikely to counteract the effect of the increased activity of hTopI in the Caco2 cells completely. Hence, other factors may act in combination with the observed differences in TDP1 activity to explain the similar CPT response of Caco2 and HT29 cells. Indeed, when hTopI activity measurement was performed in the presence of CPT a marked difference in the CPT sensitivity of hTopI from the two cell lines was detected. Since the hTopI activity measurement was performed in a divalent cation depleted buffer with an assay strictly specific to hTopI, it indicates that the hTopI enzyme from the two cell lines, Caco2 and HT29 respond differently to CPT. We did not identify any mutations that could explain this difference, suggesting that the difference could be due to posttranslational modification of the hTopI protein. Note, that we cannot rule out that different intracellular CPT accumulation may also influence the survival of the Caco2 and HT29 cell lines.

Thus, *hTOPI* gene copy number, protein amount, and even hTopI activity cannot always accurately predict CPT sensitivity. Measuring hTopI activity in the presence of CPT increases the chance of precise estimation of cellular CPT sensitivity; however additional assays, such as measurement of TDP1 activity, could be included to maximize the chance of accurately predicting the CPT response.
